# The State of Coronary Arteries in Perimenopausal Women With Chest Pain

**DOI:** 10.14740/jocmr1915w

**Published:** 2014-09-09

**Authors:** Ganna S. Isayeva

**Affiliations:** National Institute of Therapy Named After L.T. Malaya, Prospekt Postysheva 2a, Kharkov 61139, Ukraine. Email: anna_isayeva_74@yahoo.co.uk

**Keywords:** Women, Climacteric period, Perimenopause, Atherosclerosis

## Abstract

**Background:**

The goal of the study was to assess the state of coronary arteries in perimenopausal women undergoing examination before prescription of hormonal replacement therapy.

**Methods:**

One hundred ninety-three patients were screened, and 43 of them were selected for inclusion to the study. Pretest probability of coronary heart disease (CHD) was 47% for patients with typical angina pain and 20.5% with atypical pain. Patients with typical and atypical pain syndromes had no essential differences in terms of age, but had different hormonal status assessed by follicle stimulating hormone level and different menopause durations.

**Results:**

Atherosclerotic lesion causing luminal occlusion by more than 50% was detected in 13 (61.9%) patients with typical pain syndrome and in eight (36.4%) patients with atypical pain syndrome manifestations. Numbers of patients with intact coronary arteries were six (28.6%) and 10 (45.5%) in groups with typical and atypical pain syndrome, respectively. Myocardial bridges were found in five (23.8%) patients with typical pain syndrome and seven (31.8%) patients with atypical manifestations.

**Conclusion:**

In spite of modest pretest CHD probability in the group with typical pain syndrome and low pretest CHD probability in the group with atypical manifestations, patients with major atherosclerotic lesions were detected in both groups. Use of this approach to assessment of the state of coronary arteries allows detecting patients at high risk of cardiovascular complications and avoiding the use of hormonal replacement therapy in them.

## Introduction

Hormonal replacement therapy is not a recommended method for coronary heart disease (CHD) prevention. Nevertheless, hormonal replacement therapy prescription at early perimenopause and at intact coronary arteries is believed to decelerate formation of atherosclerotic lesions and to result in decreased mortality from cardiovascular diseases [1, 2]. Thus, the experts of International Menopause Society remark that hormonal replacement therapy prescription to patients aged fewer than 60 and being at their early postmenopause is safe and promotes decrease of mortality due to CHD [3]. Thus, exclusion of coronary arteries atherosclerotic lesions in perimenopausal women before prescription of hormonal replacement therapy is of utmost importance.

It should be mentioned that a medical practitioner frequently faces several challenges when diagnosing chronic forms of CHD in women. Thus, evaluation of clinical symptoms (chest pain) in female patients is much more complicated than establishing diagnosis in a man with similar complaints. Classical description of angina pain stated in medical manuals is more typical of male population, while women are more frequently encountered with pain syndrome atypical manifestations. Thus, female patients more frequently experience pain sensations unrelated with physical load, nocturnal pain and pain sensations related with emotional stress, as well as long-term dull pain invoked by physical load [4-6].

Use of stress testing does not always allow establishing the correct diagnosis as well due to its lower specificity in women compared to men [7]. Stangl et al have shown that specificity of test with metered physical load is lower in female population versus the male one (33-73% in women vs. 74-89% in men) [8]. Use of stress tests is currently limited in Ukraine; besides, topical administration of medications is recommended, which makes the testing yet less available. Ergonovin systemic exposure is not recommended, and dobutamine systemic exposure is frequently accompanied with severe adverse events [7].

In this case, a medical practitioner frequently applies angiography. One of its variants is computed tomography (CT) of coronary arteries, which more and more frequently becomes a part of routine clinical practice due to lack of invasive interventions, and is recommended as a method of choice in patients with low to moderate risk.

The goal of the current work was the study of the state of coronary arteries in perimenopausal women with pain sensations in chest.

## Materials and Methods

During the period 2011 - 2013, 194 patients referred for examination by a gynecologist before hormonal replacement therapy prescription, were examined. All patients exhibited either menstrual cycle duration changes or the absence of menstruation for not more than 5 years. Pain syndrome was classified as typical/atypical in accordance with criteria of European Society of Cardiologists (follow-up of patients with stable angina/stable coronary disease, years 2007 and 2013) [7]. Pain conforming to the three criteria listed below was classified as typical: retrosternal pain location with typical duration and pain characteristics (pressing, burning pain), clear pain relation with physical load, pain arrest at rest or after nitroglycerin dosing. Pain conforming to two criteria was classified as atypical, and pain conforming to one criterion - as non-cardiac. Patients with typical and atypical pain syndromes were exposed to multidetector (64-slice) CT-angiography of coronary arteries using an apparatus SOMATOM Definition AS, Siemens. This examination was not performed to patients with non-cardiac pain. Cholesterol and its fractions levels (low density lipoprotein cholesterol, triglycerides, high density lipoprotein cholesterol) were measured in all women. Enzymatic method was applied to determine blood lipids; measurements were carried out using Human reagents (Germany). Hormonal status of patients was assessed by follicle stimulating hormone (FSH) level. Blood was drawn during the first 5 days of menstrual cycle in patients with maintained menstrual function. In patients with absent menstruation, blood sampling was performed on any day of a month. All study subjects underwent blood sampling in fasted state at the first half of the day. Menopause was diagnosed provided FSH level was less than 30 IU/mL. FSH serum levels were measured by enzyme-linked immunoassay using a set of reagents Gonadotropin EIA-FSH from Alkor Bio Company LLC (Russian Federation).

All patients underwent treadmill stress test in accordance with Bruce’s protocol. Electrocardiographic complex with treadmill Cardio-Perfect MD (Cardio-Control, The Netherlands) was used for treadmill test.

Patients with acute coronary syndrome, surgically induced menopause, menopause lasting for more than 5 years, moderate to severe diabetes mellitus, cardiac failure of functional class III-IV, valvular heart disease, cancer pathologies, chronic obstructive pulmonary diseases, chronic renal failure of stage II and higher, thyroid gland hormone-producing function disorders, stomach and duodenal ulcers, diseases limiting the life span to 1 year, and patients with acute inflammatory processes were excluded from the study.

The study protocol was approved by Local Ethics Commission of NIT of the Academy of Medical Sciences of Ukraine; all patients were informed of the study goal and signed the informed consent for participation in the study.

The results were processed by distribution-free statistical methods of medico-biological profile using a statistical software package Excel for Windows and IBM SPSS 19.0. Null-hypothesis testing was carried out using Mann-Whitney U-test. Data were represented as median and 25% and 75% quartiles (Me (25 - 75)).

## Results

At the baseline, 193 patients were examined. Among them, pain syndrome was classified as non-cardiac pain in 141, and they were excluded from the further study. Besides, nine patients refused to undergo CT of coronary arteries. Forty-three patients were selected for angiographic examination. Patient characteristics depending on pain syndrome type are shown in Table 1.

**Table 1:** Clinical Characteristic of the Patients

	Typical chest pain (n = 21)	Atypical chest pain (n = 22)	U
Smoking, n (%)	4 (19.0%)	3 (13.6%)	0.73
Diabetes mellitus, n (%)	4 (19.0%)	2 (9.1%)	0.45
Hypertension, n (%)	17 (80.9%)	13 (58.1%)	0.28
Age, years	54.3 (48.0 - 56.5)	55.3 (38.5 - 55.9)	0.70
Menopause durations, years	4.8 (4.1 - 4.8)	3.33 (3.1 - 3.9)	0.06
FSH, IU/L	50.1 (9.0 - 68.6)	10.5 (5.9 - 69.8)	0.04
Total cholesterol, mmol/L	3.8 (3.6 - 4.9)	4.4 (3.9 - 5.0)	0.242
HDL cholesterol, mmol/L	1.0 (0.8 - 1.6)	1.1 (0.9 - 1.2)	0.558
Triglycerides, mmol/L	1.6 (1.1 - 1.9)	1.2 (1.1 - 2.0)	0.518
LDL cholesterol, mmol/L	2.1 (1.8 - 2.7)	2.5 (2.2 - 2.9)	0.202
Systolic blood pressure, mm Hg	130.0 (117.5 - 140.0)	130.0 (120.0 - 145.0)	0.205
Diastolic blood pressure, mm Hg	80.0 (77.5 - 80.0)	90.0 (70.0 - 95.0)	0.424
Heart rate, beats per minutes	67.5 (63.5 - 75.0)	75.0 (62.0 - 84.0)	0.569
Ejection fraction, %	58.0 (48.5 - 67.0)	61.5 (46.9 - 63.3)	0.959
Pretest CHD probability, %	47 (17.5 - 58.0)	20.5 (18.5 - 32.8)	0.038

Patients with typical pain syndrome had longer menopause duration (P = 0.065), and included more individuals with diabetes mellitus type 2 (P = 0.45). Differences in patients’ hormonal status were revealed. Thus, patients of atypical pain syndrome group had higher FSH level (P = 0.04). Pretest CHD probability in groups was 47% for typical pain syndrome and 20.5% for atypical one (P = 0.038).

CT-coronary angiography showed the absence of any atherosclerotic lesion of coronary arteries in 20 (45.5%) patients; four (9.1%) had atherosclerotic occlusion of less than 50% of vascular lumen; 20 (45.5%) patients exhibited atherosclerotic occlusion of more than 50% of coronary artery. Among patients with atherosclerotic lesion of coronary arteries, seven (15.9%) had more than one affected vessel. In 10 patients with unaltered coronary arteries, muscular bridges of anterior interventricular branch of the left coronary artery were detected. In two patients, the presence of muscular bridges was combined with 30% atherosclerotic occlusion of the right coronary artery.

Patients undergoing CT examination of coronary arteries were distributed into two groups depending on pain syndrome type; coronary angiography results obtained in these two groups are shown in Table 2.

**Table 2:** The State of Coronary Arteries in Patients With Typical and Atypical Pain Syndrome

	Patients with typical chest pain (n = 21)	Patients with atypical chest pain (n = 22)	Chi-squared value, P-level
Normal coronary artery	6 (28.6%)	10 (45.5%)	1.31, P = 0.25
Multiple atherosclerosis of coronary arteries	6 (28.6%)	1 (4.54%)*	4.55, P = 0.03
Atherosclerotic stenosis of less than 50%	2 (9.5%)	4 (18.2%)	0.67, P = 0.41
Atherosclerotic stenosis of more than 50%	13 (61.9%)	8 (36.4%)	2.81, P = 0.09
Myocardial bridges	5 (23.8%)	7 (31.8%)	0.34, P = 0.56
Hypoplasy of right coronary artery	0	2 (9.1%)	2.00, P = 0.16
Agatston score	160 (11.0 - 369.7)	10 (0 - 72.5)*	P = 0.011

Women with atherosclerotic stenosis of more than 50% of vascular lumen, as well as patients with multivascular lesions, reliably prevailed in the group with typical pain. It is worth mentioning that 30% atherosclerotic lesion of anterior interventricular branch of left coronary artery was combined with hypoplasia of the right coronary artery in one patient from atypical pain syndrome group. Besides, patients with typical pain syndrome had reliably higher Agatston score.

## Discussion

Atherosclerotic lesion identification before the beginning of hormonal replacement therapy is an essential step. Patients participating in this study had low scores of CHD pretest probability. Thus, CHD pretest probability is 47% for patients with typical pain in age group 50 - 59 years old, while pretest probability of this disease does not exceed 20% for patients with atypical pain of this age group. At the same time, our study has revealed hemodynamically essential atherosclerotic occlusion of coronary arteries in 61.9% patients of the first group and 36.4% patients of the second group. It should be mentioned that this is the group where hormonal replacement therapy is contraindicated to women, and doctor’s further actions are quite clear in this case.

From the examined group, 28.6% women with typical pain syndrome and 45.5% women with atypical pain syndrome had intact coronary arteries. Higher prevalence of intact coronary arteries in women was detected in such studies as CASS (coronary artery surgery study) - 39% of women [9], NCDR (American College of Cardiology-National Cardiovascular Data Registry) - 51% of female patients [10]; the maximal number of intact coronary arteries was detected in women during WISE study (Women’s Ischemic Syndrome Evaluation) - 62% [11]. In our study, patients with positive stress test result may be viewed as patients with syndrome X. Literature data regarding the effects of hormonal replacement therapy on the course of cardiac syndrome X are rather limited. A very small-scale study by Rosano et al including 25 patients has shown the decrease in frequency of angina pectoris attacks from 7.3 to 3.7 episodes in 10 days with concomitant transdermal 17-beta-estradiol administration [12]. A yet smaller study by Albertsson et al has shown improved physical load tolerability (in accordance with stress test data) and increased time to ischemia onset achieved by 17-beta-estradiol therapy of patients with cardiac syndrome X [13]. Nevertheless, in accordance with available recommendations, hormonal replacement therapy is prescribed only to patients without cardiovascular system affection; in our study, this therapy was not recommended to patients.

Interpretation of results in patients with muscular bridges is the most complicated. This pathology is inherited; nevertheless, its clinical importance is widely discussed in literature, and there is no unequivocal opinion regarding this issue [14]. We did not recommend hormonal replacement therapy to patients with muscular bridges and positive treadmill test result.

The last patient group includes women with minimal atherosclerosis manifestations. No literature data regarding the possible use of hormonal replacement therapy in them are available. In accordance with current recommendations (not to prescribe hormonal replacement therapy to patients with signs of cardiovascular system lesions), they were not recommended to use therapy with hormonal drugs.

Thus, before scheduling hormonal replacement therapy, patients need thorough examination, consideration of risk factors, and comprehensive examination of the state of coronary arteries in order to preclude adverse cardiovascular side effects.

### Study limitations

This study was conducted in a small patient cohort. This is due to the fact that only women planning hormonal replacement therapy were selected for inclusion. Besides, patients were initially referred by a gynecologist. If the study was conducted with involvement of patients referring to a cardiologist/family doctor (therapist), the level of detected coronary artery lesions could have been higher. Besides, location of atherosclerotic lesions was not performed in the study, as the investigators were first of all interested not in finding out clinical pattern conformity to certain lesion sites, but in detection of an atherosclerotic lesion as such in view of contraindications to hormonal replacement therapy.

### Conclusions

Perimenopausal patients with typical and atypical pain syndromes represent a heterogenic group in terms of the state of coronary arteries.

Among patients with low CHD pretest probability, atherosclerotic lesions were detected in more than half of women (61.9%) with typical pain syndrome, and in 36.4% women with atypical pain.

Other coronary angiography findings in this patient group included muscular bridges, right coronary artery hypoplasia and hemodynamically minor atherosclerosis of coronary arteries.

## References

[1] Rossouw JE, Prentice RL, Manson JE, Wu L, Barad D, Barnabei VM, Ko M (2007). Postmenopausal hormone therapy and risk of cardiovascular disease by age and years since menopause. JAMA.

[2] Santen RJ, Allred DC, Ardoin SP, Archer DF, Boyd N, Braunstein GD, Burger HG (2010). Postmenopausal hormone therapy: an Endocrine Society scientific statement. J Clin Endocrinol Metab.

[3] Sturdee DW, Pines A, Archer DF, Baber RJ, Barlow D, Birkhauser MH, Brincat M (2011). Updated IMS recommendations on postmenopausal hormone therapy and preventive strategies for midlife health. Climacteric.

[4] Douglas PS, Ginsburg GS (1996). The evaluation of chest pain in women. N Engl J Med.

[5] O'Keefe-McCarthy S (2008). Women's experiences of cardiac pain: a review of the literature. Can J Cardiovasc Nurs.

[6] Volkov VI, Isayeva GS (2010). Diagnostic and prognostic value of appropriate evaluation of chest pain in women. Liky Ukrainy.

[7] Montalescot G, Sechtem U, Achenbach S, Andreotti F, Arden C, Budaj A, Bugiardini R (2013). 2013 ESC guidelines on the management of stable coronary artery disease: the Task Force on the management of stable coronary artery disease of the European Society of Cardiology. Eur Heart J.

[8] Stangl V, Witzel V, Baumann G, Stangl K (2008). Current diagnostic concepts to detect coronary artery disease in women. Eur Heart J.

[9] Kemp HG, Kronmal RA, Vlietstra RE, Frye RL (1986). Seven year survival of patients with normal or near normal coronary arteriograms: a CASS registry study. J Am Coll Cardiol.

[10] Akhter N, Milford-Beland S, Roe MT, Piana RN, Kao J, Shroff A (2009). Gender differences among patients with acute coronary syndromes undergoing percutaneous coronary intervention in the American College of Cardiology-National Cardiovascular Data Registry (ACC-NCDR). Am Heart J.

[11] Khuddus MA, Pepine CJ, Handberg EM, Bairey Merz CN, Sopko G, Bavry AA, Denardo SJ (2010). An intravascular ultrasound analysis in women experiencing chest pain in the absence of obstructive coronary artery disease: a substudy from the National Heart, Lung and Blood Institute-Sponsored Women's Ischemia Syndrome Evaluation (WISE). J Interv Cardiol.

[12] Rosano GM, Peters NS, Lefroy D, Lindsay DC, Sarrel PM, Collins P, Poole-Wilson PA (1996). 17-beta-Estradiol therapy lessens angina in postmenopausal women with syndrome X. J Am Coll Cardiol.

[13] Albertsson PA, Emanuelsson H, Milsom I (1996). Beneficial effect of treatment with transdermal estradiol-17-beta on exercise-induced angina and ST segment depression in syndrome X. Int J Cardiol.

[14] Donkol RH, Saad Z (2013). Myocardial bridging analysis by coronary computed tomographic angiography in a Saudi population. World J Cardiol.

